# Mesencephalic Astrocyte-Derived Neurotrophic Factor Regulates Morphology of Pigment-Dispersing Factor-Positive Clock Neurons and Circadian Neuronal Plasticity in *Drosophila melanogaster*

**DOI:** 10.3389/fphys.2021.705183

**Published:** 2021-09-27

**Authors:** Wojciech Krzeptowski, Lucyna Walkowicz, Ewelina Krzeptowska, Edyta Motta, Kacper Witek, Joanna Szramel, Terence Al Abaquita, Zbigniew Baster, Zenon Rajfur, Ezio Rosato, Vassilis Stratoulias, Tapio I. Heino, Elżbieta M. Pyza

**Affiliations:** ^1^Department of Cell Biology and Imaging, Institute of Zoology and Biomedical Research, Jagiellonian University, Kraków, Poland; ^2^Faculty of Physics, Astronomy and Applied Computer Science, Jagiellonian University, Kraków, Poland; ^3^Jagiellonian Center of Biomedical Imaging, Jagiellonian University, Kraków, Poland; ^4^Department of Genetics, University of Leicester, Leicester, United Kingdom; ^5^Molecular and Integrative Biosciences Research Program, Faculty of Biological and Environmental Sciences, University of Helsinki, Helsinki, Finland; ^6^Neuroscience Center, HiLIFE, University of Helsinki, Helsinki, Finland

**Keywords:** circadian rhythms, circadian clock, visual system, locomotor activity, neurotrophic factor

## Abstract

Mesencephalic Astrocyte-derived Neurotrophic Factor (MANF) is one of a few neurotrophic factors described in *Drosophila melanogaster* (DmMANF) but its function is still poorly characterized. In the present study we found that DmMANF is expressed in different clusters of clock neurons. In particular, the PDF-positive large (l-LNv) and small (s-LNv) ventral lateral neurons, the CRYPTOCHROME-positive dorsal lateral neurons (LNd), the group 1 dorsal neurons posterior (DN1p) and different *tim*-positive cells in the fly’s visual system. Importantly, DmMANF expression in the ventral lateral neurons is not controlled by the clock nor it affects its molecular mechanism. However, silencing *DmMANF* expression in clock neurons affects the rhythm of locomotor activity in light:dark and constant darkness conditions. Such phenotypes correlate with abnormal morphology of the dorsal projections of the s-LNv and with reduced arborizations of the l-LNv in the medulla of the optic lobe. Additionally, we show that DmMANF is important for normal morphology of the L2 interneurons in the visual system and for the circadian rhythm in the topology of their dendritic tree. Our results indicate that DmMANF is important not only for the development of neurites but also for maintaining circadian plasticity of neurons.

## Introduction

The fruit fly (*Drosophila melanogaster*) is a valuable model species in neuroscience to study the development of the nervous system, the molecular basis of behaviour, circadian rhythms and to model human neurodegenerative diseases (Bellen et al., 2010). A simple behavioural assay to analyse circadian rhythms is recording locomotor activity (Rosato and Kyriacou, 2006). This approach allowed to recognize specific neuronal clusters responsible for the regulation of circadian rhythms in behaviour. These so-called pacemaker neurons or master clock neurons generate rhythms that are synchronized to cyclic environmental changes. In the fly’s brain approximately 150 pacemaker neurons have been identified, which form a few clusters: the small ventral lateral neurons (s-LNvs), the large ventral lateral neurons (l-LNvs), the dorsal lateral neurons (LNds), the lateral posterior neurons (LPNs), and three groups of dorsal neurons (DN1, DN2, and DN3) (Helfrich-Förster, 2005). The clock neurons are characterized by the rhythmic expression of clock genes and their proteins. Four core clock proteins; PERIOD (PER), TIMELESS (TIM), CLOCK (CLK), and CYCLE (CYC), form transcription/translation feedback loops. In the first negative feedback loop, the CLK/CYC heterodimer activates transcription of the *tim* and *per* genes. Next, TIM and PER proteins accumulate in the cytoplasm, form heterodimers and then enter the nucleus to inhibit the activity of CLK/CYC and the expression of their own genes. A new cycle begins when TIM and then PER are degraded and CLK/CYC-dependent transcription starts again. In the second positive feedback loop, VRILLE (VRI) and PAR DOMAIN PROTEIN 1 ε (PDP1ε) regulate *Clk* transcription, which strengthens circadian molecular oscillations. As these two loops share common elements they are interlocked. Noteworthy, post-transcriptional and post-translational processes also contribute to the molecular mechanism of the clock (reviewed in Özkaya and Rosato, 2012).

The fly’s pacemaker neurons form a highly plastic network, which can adapt to changes in the environment and regulate cellular and biochemical pathways throughout the day. Among signalling molecules, neuropeptides and fast neurotransmitters have been identified as crucial factors in the fly’s circadian system (reviewed in Beckwith and Ceriani, 2015). Special attention has been paid to the neuropeptide PIGMENT-DISPERSING FACTOR (PDF) expressed by the l-LNvs and four of the five s-LNvs (Kaneko et al., 1997) and released in a circadian fashion (Park et al., 2000). PDF is crucial for the normal rhythmicity of flies under constant conditions (Renn et al., 1999) and is required for synchronization of different groups of clock neurons in the brain (Lin et al., 2004). The PDF-expressing s-LNvs seem crucial for generating the morning peak of locomotor activity under light:dark conditions (LD) and rhythmicity in constant darkness (DD) and they are called M-cells (morning oscillators or main oscillators) (Grima et al., 2004; Rieger et al., 2006; Shafer and Taghert, 2009). In turn, the evening peak of activity is regulated by the PDF-negative 5th s-LNv and the three cryptochrome-expressing LNds, which are E-cells (Cusumano et al., 2009). On the other hand, it has been shown that large PDF-positive neurons also regulate flies’ behaviour since they are involved in light-mediated arousal and contribute to the timing of evening activity under long days (Shang et al., 2008; Schlichting et al., 2019).

In the nervous system, neurotrophic factors (NTFs) regulate various processes including cell differentiation, survival of neurons, neural plasticity, and neurite outgrowth (Loughlin and Fallon, 1993). An increasing body of evidence indicates that they are also implicated in the regulation of circadian pacemaker functions in mammals. For instance, Brain-Derived Neurotrophic Factor (BDNF) shows circadian expressaion in rat clock neurons, with higher levels corresponding to increased locomotor activity and body temperature (Liang et al., 1998; Naert et al., 2006). Conversely, Transforming Growth Factor alpha (TGF-α) that is rhythmically expressed in the brain of mice, inhibits locomotion (Naert et al., 2006). In *Drosophila* the best characterized neurotrophic factor is Mesencephalic Astrocyte-derived Neurotrophic Factor (DmMANF), that shares approximately 50% amino acid sequence identity to its mammalian orthologues Mesencephalic Astrocyte-derived Neurotrophic Factor (MANF) and Cerebral Dopamine Neurotrophic Factor (CDNF) (Palgi et al., 2009). This neurotrophic factor seems to be especially crucial for dopaminergic neurons in both rodents and flies. The lack of MANF decreases the number of dopaminergic neurons during zebrafish embryonic development (Chen et al., 2012) while in *Drosophila* it leads to the degeneration of dopaminergic neuron axons and reduced dopamine level (Palgi et al., 2009). Moreover, MANF treatment in rat model of Parkinson’s disease protects and restores dopaminergic neurons (Voutilainen et al., 2009). Numerous *in vitro* and *in vivo* experiments have also demonstrated that MANF/DmMANF has protective and immunomodulatory capacity and its expression is upregulated in response to endoplasmic reticulum (ER) stress (Mizobuchi et al., 2007; Apostolou et al., 2008; Yu et al., 2010; Palgi et al., 2012; Shen et al., 2012; Lindahl et al., 2014; Lindström et al., 2016; Neves et al., 2016).

In this study, we aimed to determine if DmMANF may be important for the development of clock cells, in particular the PDF-positive clock neurons, and for neuronal plasticity. We found that DmMANF is expressed in three major clock neuron clusters: the large and small PDF-positive ventral lateral neurons, the CRYPTOCHROME-positive dorsal lateral neurons and the dorsal neurons DN1p. Moreover, RNAi-induced knock-down of DmMANF in clock cells affected the morphology of the projections of the PDF-expressing LNvs, which in turn generate circadian rhythms in flies. In the visual system, we also observed that lack of DmMANF in the L2 interneurons of the lamina, which show circadian plasticity (Pyza and Meinertzhagen, 1999; Weber et al., 2009), abolished the rhythmic size change of their dendritic tree.

## Materials and Methods

### Fly Stocks

Flies were reared on a standard corn/yeast medium at 25°C in a 12 h:12 h light:dark cycle (LD 12:12), unless otherwise noted. Canton S was employed as a wild type strain and *w*^1118^ (a kind gift of F. Rouyer, Paris-Saclay Institute of Neuroscience, CNRS, France) was crossed to GAL4 and UAS lines to generate parental controls. The following strains were obtained from the Bloomington *Drosophila* Stock Centre (BDSC): *UAS-mCD8::GFP* (# 5137), *UAS-S65T-GFP* (# 1521). The following stocks were obtained from colleagues or were already available in the laboratory: *UAS-DmMANF-RNAi*, *UAS-DmMANF-RNAi UAS-Dicer2, UAS-Dicer2* (Stratoulias and Heino, 2015b), *UAS-rpl-GFP* (BDSC #42682 from K. Jagla, GReD, University of Clermont Auvergne), *tim-GAL4* (more about the pattern of *tim-GAL4* expression in Kaneko and Hall, 2000), *clk4.1M-GAL4* (yw;;clk gal4 4.1M/TM6B; expression in 8–10 DN1s) and *mai^179^-GAL4* (expression in three CRY-positive LNds, four PDF-positive s-LNvs and in PDF-negative 5th s-LNv) (from F. Rouyer, Paris-Saclay Institute of Neuroscience, CNRS), *Pdf-GAL4* (expression in PDF-positive neurons; four l-LNvs and four s-LNvs, including their projections), *w; EGUF/+; FRT80B GMR-hid3L/TM6B* [(Stowers and Schwarz, 1999) from S. Bartoszewski, University of Rzeszów], *elav-GAL80, repo-GAL80, Pdf-GAL80* (from C. Wegener, University of Würzburg), *21D-GAL4* (expression in L2 cells; from T. Raabe, University of Würzburg).

### Mosaic Analysis With the MARCM System

To label single L2 neurons (identified by the driver line *21D-GAL4*) which lack *DmMANF*, we used the Mosaic Analysis with a Repressible Cell Marker (MARCM) system for clonal analysis. Using standard genetics we obtained line A (+/+; *UAS-mCD8::GFP*/*UAS-mCD8::GFP*; *21D-GAL4 FRT 82B DmMANF^Δ96^*/*Tm6 Tb Sb)* and line B (*hsFLP*/*hsFLP; +/+; FRT 82B tubP-GAL80/FRT 82B tubP-GAL80)*. After crossing, their progeny were heat shocked at 37°C for 1 h every 24 h during the whole larval development until pupation. In the progeny, only homozygous mutant clones (*hsFLP*/+ (Y); *UAS-mCD8::GFP*/+; *21D-GAL4 FRT 82B DmMANF^Δ96^*/ *21D-GAL4 FRT 82B DmMANF^Δ96^*) are labelled with GFP.

### Western Blot

Males (7–10 days old) were frozen in liquid nitrogen at ZT1, ZT4, ZT13, and ZT16 (ZT is Zeitgeber Time, where ZT0 = lights-on and ZT12 = lights-off) or at CT1, CT4, CT13, and CT16 (CT is Circadian Time, where CT0 = the beginning of subjective day and CT12 = the beginning of subjective night). First, flies were kept under LD 12:12 for 4 days of their life, and then placed under DD for three consecutive days, and after they were fixed for analyses. Heads were dissected on ice, transfered into microcentrifuge tubes and mechanically crushed in liquid nitrogen with disposable plastic pestles. Tissues were homogenized by sonication using an ultrasonic homogenizer (UP-100H, Hielscher) in 2 × Laemmli buffer with protease inhibitors (Boehringer). Next, samples were placed on ice, gently shaken on a horizontal shaker, frozen at −20°C, thawed and centrifuged at 13,200 rpm for 1 h at 4°C. Finally, the supernatant was denatured at 80°C for 5 min before loading. Protein concentrations were measured using the Micro BCA Protein Assay Kit (Thermo Scientific) measuring absorbance at 570 nm on a plate reader (Spectramax iD3, Molecular Devices). Electrophoresis were performed on a NuPAGE SDS-PAGE Gel System using pre-cast 4–12% Bis-Tris gel (Thermo Scientific) gradient gels. 100 μg of protein were loaded per sample. Proteins were blotted with a iBlot 2 Dry Blotting System onto a PVDF membrane (Thermo Scientific^*TM*^) followed by blocking with 5% dry milk in PBST (phosphate buffer saline, 0.1% Tween-20) for 1 h at room temperature. Membranes were incubated with rabbit anti-DmMANF [1:5000; (Palgi et al., 2009)] serum and anti-α tubulin (1:20000, ab4074, Abcam) as a loading control. As secondary antibody, we used goat anti-rabbit HRP (1:10000, ab6721, Abcam). Immunodetection was carried out by ECL (PerkinElmer) and densitometric quantification was performed using ImageJ software. Western blot analyses were repeated at least three independent times.

### Immunohistochemistry

For analysis of L2 neuron morphology, one-week-old males were decapitated at ZT1, ZT4, ZT13, and ZT16 and fixed in 4% paraformaldehyde (PFA) in 0.1 M phosphate buffer for 3 h at 4°C. Thereafter, heads were washed in phosphate buffer saline (PBS), cryoprotected overnight at 4°C in 25% sucrose and embedded in Shandon Cryomatrix embedding resin (Thermo Scientific). Cryosections, 20 μm thick, were immunolabelled with primary antibodies at 4°C overnight and then with secondary antibodies at 4°C overnight as well. Finally, brain sections were washed and mounted in Vectashield medium (Vector Laboratories).

For whole brain immunolabelling, heads of 1-week-old males were fixed in 4% PFA in PBS with 0.1% Triton X 100 (0.1% PBS-Tx) for 3 h at room temperature at ZT1 (to check colocalization of DmMANF with PDF or to visualize morphology of PDF- and MIP-positive neuron projections) at ZT2, ZT14 and CT2, CT14 (CT is Circadian Time, the subjective time under constant conditions with CT0 = the beginning of the subjective day and CT12 = the beginning of the subjective night) to analyse the expression of PER. Next, flies were washed twice in PBS for 15 min and once in PBS with 0.5% Triton X 100 (0.5% PBS-Tx) for 15 min. Afterward, brains were dissected, blocked overnight with 5% normal goat serum in 0.5% PBS- Tx and immunolabelled with primary antibodies at 4°C overnight. The brains were then washed three times in 0.5% PBS-Tx for 15 min and incubated with the secondary antibody at 4°C overnight. Finally, brains were washed and mounted in Vectashield medium. Images were taken using a Zeiss LSM 710 confocal microscope.

The following primary antibodies were used in the study: rabbit anti-DmMANF [1:1000, (Palgi et al., 2009)], mouse anti-PDF (1:500, Developmental Studies Hybridoma Bank- DSHB), mouse anti-ELAV (1:50, DSHB), mouse or rabbit anti-GFP (1:1000, Novus Biologicals), anti-PER (1:1000, a kind gift of R. Stanewsky, University of Münster), rabbit anti-MIP [1:250, a kind gift of C. Wegener, University of Würzburg, (Predel et al., 2001)]. The secondary antibody used were as follows: goat anti-rabbit (1:400, Jackson ImmunoResearch Laboratories) Cy3-conjugated and goat anti-mouse (1:1000, Molecular Probes Invitrogen) Alexa Fluor 488- conjugated antibodies or goat anti-mouse (1:400, Jackson ImmunoResearch Laboratories) Cy3- conjugated.

### Sholl Method of Neuronal Branch Analysis

To quantify axonal arbors of s-LNvs in the dorsal protocerebrum and neurites in the second optic neuropil (medulla) of PDF-positive neurons (l-LNvs) we used the Sholl method described by Fernández et al. (2008). We used the plug-in “Sholl analysis” [v 3.6.12, (Ferreira et al., 2014)] in the Fiji software. As starting point (rings were plotted every 10 μm) we selected the position of the first neuronal branch and we counted the number of intersections of each branch with a given ring. We compared the total number of axonal crosses across genotypes.

### L2 Dendritic Tree Analysis

To measure the L2 dendritic tree perimeter, we used *21D > mCD8::GFP* (as control) and MARCM flies. Sections of the distal lamina were examined using a Zeiss Meta 510 Laser Scanning Microscope. Images were deconvolved using Huygens Professional software (Scientific Volume Imaging). Changes in the perimeter of L2 dendritic trees were examined by tracing the outline of dendrites and axons of L2 cell cross-sections. Measurements were performed using ImageJ software.

### Quantification of the Number of Pigment-Dispersing Factor-Positive Cell Bodies

Whole-mount 7 days old adult brains were labeled with anti-PDF serum and next they were examined using a Zeiss Meta 510 Laser Scanning Microscope. PDF-labeled cells were counted manually through all *z*-stacks for both hemispheres. The mean number of cells per cluster and per hemisphere was calculated.

### Locomotor Activity Analysis

Locomotor activity and sleep of the experimental flies were recorded using the *Drosophila* Activity Monitoring System 2 (DAM2, Trikinetics). Young males (1–3-days-old) were placed into glass tubes with a small amount of medium (water, sugar, and agar) and inserted into DAM2 monitors housed in an incubator under constant temperature (25°C) and humidity Locomotor activity was recorded individually over 7 days under LD12:12 and then for seven additional days under DD. The raw data after recording locomotor activity of flies were analysed using ShinyR-DAM on-line software (Cichewicz and Hirsh, 2018) and MS Excel. To generate average-activity plot events were summed up into 30-min bins that were averaged for all individuals of a given genotype and condition. Locomotor activity periods were determined by chi-square periodogram analysis and the strength of the rhythm was defined as the highest Qp.act/Qp.sig ratio. Last 6 days of DD were analysed to evaluate period and strength of the circadian rhythm of locomotor activity.

### Statistics

Experimental data were checked for the presence of outliers by the Grubbs test. Next, depending on a Shapiro-Wilk *W* test result, one–way analysis of variance (ANOVA) followed by a Tukey *post-hoc* test or Kruskal-Wallis non-parametric test, followed by multiple comparison Dunn *post hoc* test were used to estimate significant differences between groups. “*N*” represents the number of individuals. *P-*values < 0.05 were considered statistically significant. Statistical analyses of the data were carried out using Origin 2020 (OriginLab) computer software.

## Results

### *Drosophila melanogaster* Mesencephalic Astrocyte-Derived Neurotrophic Factor Is Present in the Pacemaker Neurons and *Tim*-Expressing Cells in the Medulla

In the brain of adult flies, DmMANF is expressed in glial cells and neurons (Stratoulias and Heino, 2015a; Walkowicz et al., 2017, 2021). However, approximately 50% of this protein seems to be concentrated in the retina (Supplementary Figure 1).

**FIGURE 1:**
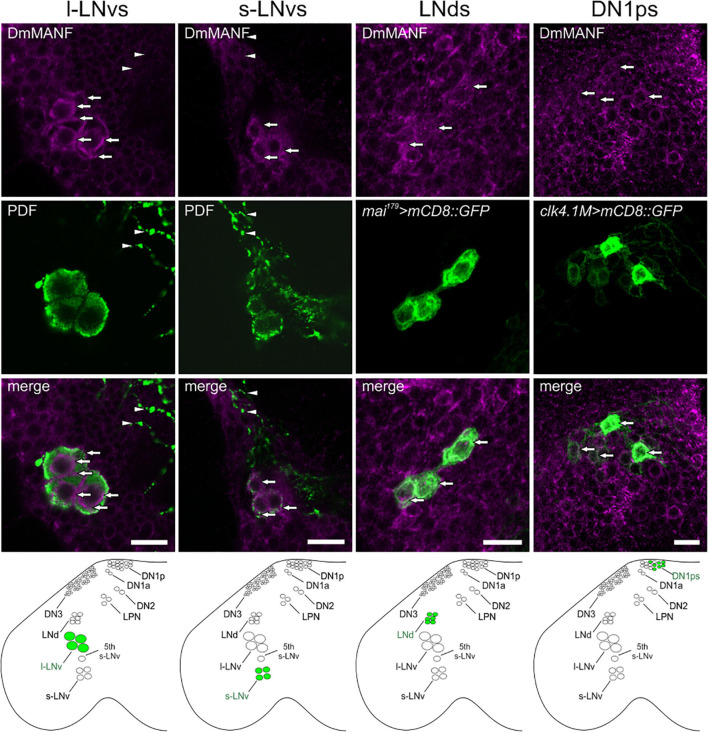
DmMANF immunolocalization in the *Drosophila* pacemaker neurons. Co-labeling of DmMANF (magenta) and different clock cells (green) in the fly’s brain whole-mount. For visualization of PDF-positive l-LNv and s-LNv neurons (*N* > 100), brains of Canton S flies were stained with anti-PDF serum. LNds and DN1ps were labeled with GFP-expression driven by *mai^179^-GAL4* (*N* = 10) and *clk4.1M-GAL4* (*N* = 10), respectively. Lower panel shows the schematic localization of the examined clusters of clock neurons in the fly brain. Arrows indicate expression of DmMANF in clock neurons. Arrowheads show l-LNv projections in the medulla and the lack of co-localisation with DmMANF. Scale bars: 5 μm.

To investigate if DmMANF is important for clock cells we examined co-localization of this protein with different markers of pacemaker cells. Double labelling with antibodies against DmMANF and PDF showed high level of DmMANF in ventral lateral neurons, both l-LNvs and s-LNvs, in Canton S flies. In the l-LNvs DmMANF localizes beneath the cell membrane and in the perinuclear region while in the s-LNvs it is diffused in cytoplasm. However, DmMANF was not detected in projections of the l-LNvs in the medulla (Figure 1) and of the s-LNvs in the dorsal brain (data not shown). Additionally, we detected DmMANF in DN1ps (*clk4.1M* > *mCD8::GFP*) and in the CRYPTOCHROME-positive LNds (*mai*^179^ > *mCD8::GFP*) (Figure 1).

To examine whether the expression of DmMANF is under clock-control, we assayed its level in whole head homogenates by Western blot. Wild type Canton S flies were collected at four time points in LD12:12 (ZT1, 4, 13, 16) and DD (CT1, 4, 13, 16). We observed weak and statistically not significant changes in the level of DmMANF under both entrainment (Supplementary Figure 2A; Kruskal-Wallis Test, *p* = 0.543) and constant (Supplementary Figure 2B; Kruskal-Wallis Test, *p* = 0.063) conditions. Whole head homogenates contain proteins from different tissues. Thus, we assayed DmMANF levels specifically in the cell bodies of PDF-expressing clock neurons (Supplementary Figure 2C). Again, we observed no statistically significant changes in the expression of DmMANF across four different time points (ZT1, 4, 13, 16) in both s-LNvs and l-LNvs (Kruskal Wallis, *p* = 0.357, *N* = 115 and *p* = 0.377, *N* = 120, respectively).

**FIGURE 2:**
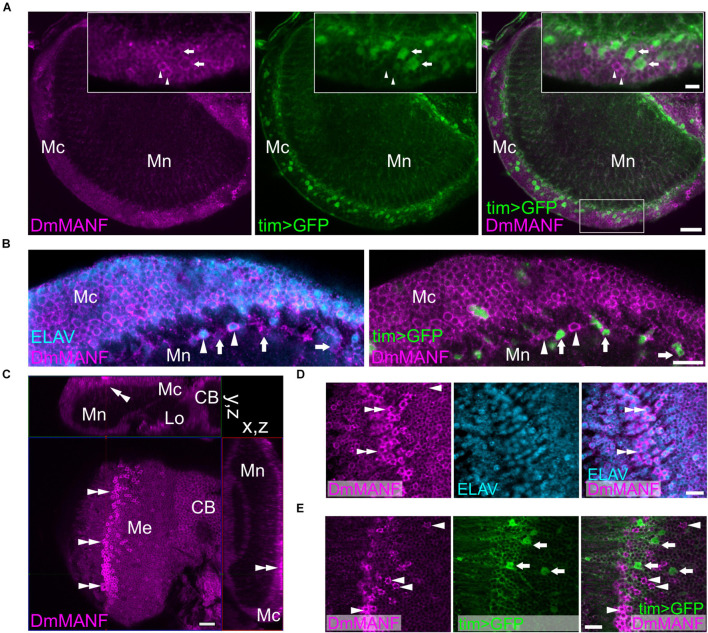
DmMANF expression in the second optic neuropil (medulla). **(A)** DmMANF is present in the majority of *tim-GAL4* driven *UAS-S65T-GFP* cells in the second optic neuropil, including cell bodies in the medulla cortex and processes in the medulla neuropil. Inserts show enlargements of the medulla cortex. Arrowheads indicate cells with high level of DmMANF and low expression of *tim*. Arrows show cells which express *tim* at high level but DmMANF at low level. **(B)** The majority of DmMANF-positive cells in the medulla cortex represents neurons (labeled with anti-ELAV, a pan-neuronal marker). These neurons also exhibit low level of nuclear UAS-rpl-GFP under *tim-GAL4* control. Similarly to the medulla cortex, in the distal part of the medulla neuropil two populations of cells, characterized by high or low levels of DmMANF, were identified. Cells strongly expressing DmMANF are neurons, as they co-expressed ELAV (arrowheads), while a population of cells with low level of DmMANF does not express ELAV but strongly expresses *tim* (arrows). **(C)** The cluster of DmMANF-positive cells was also identified on the surface of the medulla (double arrowheads). The figure represents orthogonal view of the optic lobe. **(D)** Cells with high levels of DmMANF (arrows) express ELAV pan-neuronal marker (double arrowheads) **(E)** but do not co-localize with cells which express strong mCD8::GFP signal under *tim-GAL4* control (arrows). Mc: medulla cortex, Mn: medulla neuropil, Lo: lobula complex, CB: central brain. Scale bars: 20 μm **(A,C)**, 10 μm **(B,D,E)**, 5 μm (insert in **A**). *N* = 7.

In the second optic neuropil, the medulla, we observed DmMANF in the majority of *tim*-expressing cells but following a complex correspondence (Figure 2). In the medulla cortex, we identified cell bodies with high expression of DmMANF and high expression of GFP (*tim > S65T-GFP*). However, these two cell populations did not co-localise (Figure 2A). Double labelling with DmMANF and the neuronal marker ELAV, showed that in the medulla cortex the majority of DmMANF-positive cells are neurons (Figure 2B). In the medulla neuropil we identified *tim-* and DmMANF*-*positive cells which were ELAV negative (Figure 2B). Finally, in the very distal medulla cortex we recognized DmMANF-immunoreactive cells (Figure 2C). These were also identified as neurons (Figure 2D) with a moderate expression of *tim* (Figure 2E).

### Silencing of *Drosophila melanogaster* Mesencephalic Astrocyte-Derived Neurotrophic Factor Expression in Clock Neurons Affects Rhythmic Locomotor Activity

To determine whether DmMANF is involved in the regulation of locomotor activity rhythm in flies, we decreased its expression using *UAS-RNAi* in all clock cells (neuronal and not) using the *tim-GAL4* driver (Kaneko and Hall, 2000) together with *UAS-Dicer2* or not.

First, we verified the efficiency of DmMANF silencing by immune blot quantification in whole head homogenates (Supplementary Figure 3). We observed reduced levels of DmMANF in both *tim* > *DmMANF-RNAi* and *tim* > Dm*MANF-RNAi Dcr2* flies compared to controls. Noteworthy, overexpression of Dicer2 alone under *tim-GAL4* control also reduced the level of DmMANF.

**FIGURE 3:**
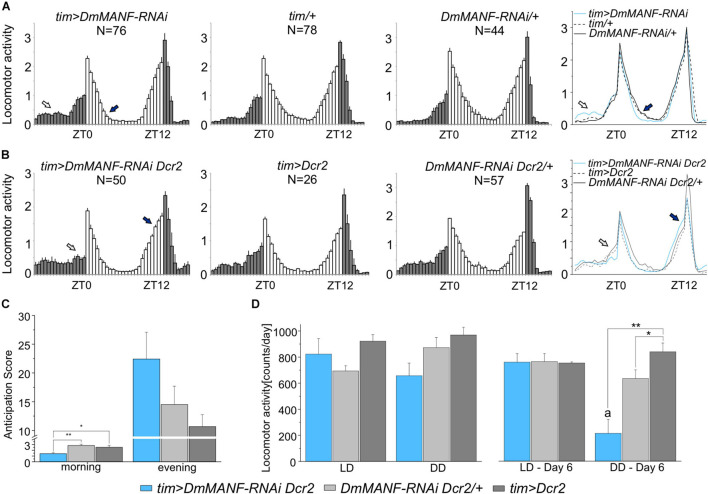
Locomotor activity in LD 12:12 and DD of flies with *DmMANF* silencing in *tim*-expressing cells. **(A)** Mean activity profiles (averaged counts per minute) in LD12:12 of *tim* > *DmMANF-RNAi* in comparison to control lines. The open arrow shows increased night-time activity while the solid arrow indicates a faster decrease in the morning peak. **(B)** Mean activity profiles (averaged counts per minute) in LD12:12 of *tim-GAL4* driven *UAS-DmMANF-RNAi UAS-Dicer2* flies in comparison to control lines. The open and solid arrows indicate lack of morning anticipation and robust evening anticipation, respectively. *N* = number of individuals; grey and white bars indicate the dark and the light phases of LD, respectively; mean ± SEM. **(C)** Morning and evening anticipation score in *tim* > *DmMANF-RNAi Dcr2* flies and controls in LD 12:12. Morning anticipation score (1.41 ± 0.2) of DmMANF-deficient flies was significantly lower when compared to *tim* > *Dcr2* (2.83 ± 0.2; *p* = 0.01) and +/Dm*MANF-RNAi Dcr2* (2.52 ± 0.3; *p* = 0.032) controls. One-way ANOVA followed by Bonferroni *post hoc* test: * *p* < 0.05, ** *p* ≤ 0.01; means ± SEM. **(D)** Mean locomotor activity (count/day) of *tim-GAL4* driven *UAS-MANF-RNAi UAS-Dicer2* flies for mean values of overall 6 days of the experiment in LD 12:12 and DD (graphs on the left) and calculated only for the sixth day in LD 12:12 or DD (graphs on the right). The activity of those flies on the sixth day of DD was significantly lower (216 ± 93) when compared to *tim* > *Dcr2* (640 ± 67, *p* = 0.023) and +/*DmMANF-RNAi Dcr2* (847 ± 68, *p* = 0.003) controls - one-way ANOVA followed by Bonferonni’s *post hoc* test. Moreover, the activity of *tim* > *DmMANF-RNAi Dcr2* flies on the sixth day in DD was also significantly lower than on the sixth day in LD 12:12 (766 ± 57, *p* = 0.004) - *t*-student Test. * *p* < 0.05, ** *p* ≤ 0.01, a – *p* < 0.05 between activity in the sixth day of DD and the sixth day of LD12:12; means ± SEM.

Next, we tested locomotor activity rhythms. Under LD 12:12 conditions, wild-type flies exhibit a bimodal pattern of activity with a morning peak around lights-on and an evening peak around lights-off. When flies are moved to DD the morning peak progressively weakens and disappears (Helfrich-Förster, 2000). The examination of activity histograms under LD 12:12 in *tim > DmMANF-RNAi* flies showed an increase in late night activity and a faster decrease of the morning activity peak (Figure 3A). When *UAS-Dicer2* was used to enhance the RNAi effect, flies did not show morning anticipation in LD 12:12 (Figure 3B), as confirmed by an anticipation score (Figure 3C) calculated as the ratio between mid-night activity (ZT18-ZT19) and late-night activity (ZT23-ZT0, just before lights-on) (ANOVA, *p* = 0.007). Furthermore, *tim* > Dm*MANF-RNAi Dcr2* flies showed higher evening activity (Figure 3B) and slightly higher evening anticipation, calculated as the activity ratio ZT11-12/ZT6-7 (Figure 3C; ANOVA, *p* = 0.146). Actograms of *tim* > Dm*MANF-RNAi Dcr2* flies are presented in the Supplementary Figure 4.

Analyses of average activity profiles of experimental groups and relevant controls in LD 12:12 did not show any effect of DmMANF silencing (Figure 3D). Total activity levels as well as day-time and night-time activity were similar in experimental and control flies (data not shown). In DD *tim* > *DmMANF-RNAi Dcr2* flies showed a small and not significant decrease of mean activity (Figure 3D; ANOVA, *p* = 0.068). However, we observed pronounced differences on the sixth day of DD (Figure 3D; ANOVA, *p* = 0.002). The mean activity of *tim > DmMANF-RNAi Dcr2* flies was significantly lower than both controls. The activity of these files was significantly lower on the sixth day of DD than on the sixth day of LD 12:12 (Figure 3D; *t*-test, *p* = 0.004).

Moreover, we found that inhibition of *DmMANF* expression by the RNAi construct alone (ANOVA, *p* < 0.001) or in combination with *Dicer2* (ANOVA, *p* = 0.002) resulted in lengthening of the period of the free-running rhythm by approximately 1 h in comparison to controls. Finally, *tim* > *DmMANF-RNAi Dcr2* individuals were more arrhythmic (13%) and showed higher mortality these differences were not statistically significant, however (Table 1).

**TABLE 1:** Period (tau) and strength of the rhythm under DD in flies subjected to downregulation of DmMANF in clock cells.

**Genotype**	**Tau ± SEM**	**Strength ± SEM**	**% *A***	**% Dead**	***N***
*tim > DmMANF-RNAi*	24.80.16^ab^	1.67 ± 0.09	1	0	76
*tim/+*	24.00.03	2.16 ± 0.11	0	1	79
*+/DmMANF-RNAi*	23.80.06	1.86 ± 0.23	0	6	47
*tim > DmMANF-RNAi Dcr2*	24.90.26^cde^	1.39 ± 0.13	13	16	56
*tim > Dcr2*	24.10.12	1.62 ± 0.03	0	4	28
*+/DmMANF-RNAi Dcr2*	23.80.05	1.82 ± 0.10	0	2	58

*The expression of *DmMANF* was reduced using the *tim-GAL4* driver.*

*For each genotype the period (tau) and the strength of the rhythm were averaged across all rhythmic flies.*

*One-way ANOVA with Bonferroni *post-hoc* test: a: *p* = 0.001 in comparison with *tim*/+, b: *p* < 0.001 in comparison with +/*DmMANF-RNAi*, c: *p* = 0,04109 in comparison with tim > Dcr2, d: *p* = 0,00305 in comparison with +/*DmMANF-RNAi Dcr2*, e: *p* = 0,00587 in comparison with tim/+.*

**A*- arrhythmic flies, *N*- number of flies tested.*

In summary these results indicate that silencing *DmMANF* expression in clock cells affects the rhythm of locomotor activity. In LD12:12 DmMANF knockdown disrupts morning anticipation. In DD, it leads to a decrease of total activity and lengthening of the free-running period.

### *Drosophila melanogaster* Mesencephalic Astrocyte-Derived Neurotrophic Factor Knockdown in *Tim*-Expressing Cells Affects the Morphology of the Pigment-Dispersing Factor-Expressing Neurons but Does Not Change the Oscillation of the PERIOD Protein

**FIGURE 4:**
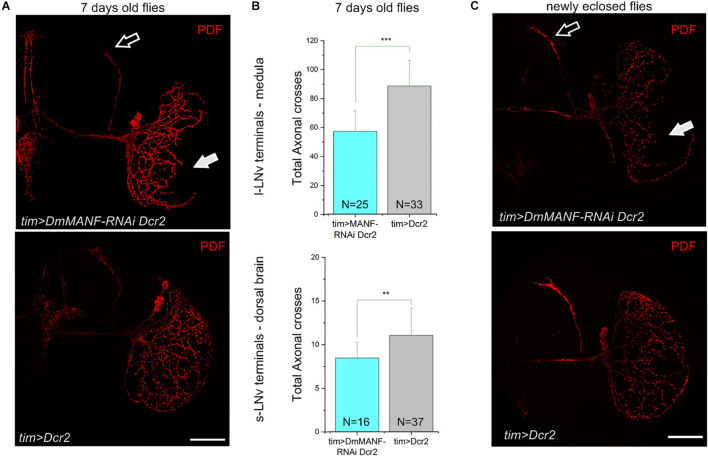
Reduced DmMANF expression affects the morphology of the PDF-positive cells. **(A)** PDF immunostaining of the projections of the s-LNvs in the dorsal brain (open arrow) and of the l-LNvs in the medulla (solid arrow). Flies with reduced DmMANF expression (*tim* > *DmMANF-RNAi Dcr2*) are compared to control (*tim* > *Dcr2*). **(B)** Sholl analysis shows reduced total axonal crosses in *tim* > *DmMANF-RNAi Dcr2* flies compared to *tim* > *Dcr2* flies. This is true for both l-LNvs (56.75 ± 14.29 *vs*. 88.22 ± 17.72; *t*-test: *p* < 0.001) and s-LNvs (8.44 ± 1.8 *vs*. 11.03 ± 3.1, Mann-Whitney Test: *p* = 0.0015). *N* represents the number of individuals used for each analysis. **(C)** The same morphological changes are observed in newly eclosed flies (few hours after eclosion). Scale bars: 50 μm.

The rhythmic changes observed in DmMANF deficient flies may originate from central clock neurons. Thus we examined the morphology of the l-LNvs and the s-LNvs including their projections to the medulla and to the dorsal brain, respectively. The most severe phenotype was observed when the *UAS-DmMANF-RNAi* construct was co-expressed together with Dicer2. In these flies we observed shorter arborizations of the l-LNvs to the medulla than in controls (Figure 4A). To quantify the l-LNv arborizations we used an adaptation of the Sholl’s method (Fernández et al., 2008). As expected, the total number of intersections was significantly lower in *tim* > *DmMANF-RNAi Dcr2* flies than in *tim* > *Dcr2* controls (Figure 4B). Reduced projections of the l-LNv to the distal medulla are likely caused by a developmental defects and not by degeneration during adult life. In fact, we observed the same defects in newly eclosed flies (Figure 4C). Moreover, the number of cell bodies was the same in experimental and control groups (Supplementary Figure 5; Mann-Whitney Test, *p* = 0.15). Likewise, Sholl’s analysis of *tim* > *DmMANF-RNAi Dcr2* flies showed that the dorsal projections of the s-LNv were less branched (Figure 4A) and that the number of total axonal crosses (Figure 4B) was significantly lower than in controls. We did not detect similar morphological changes in *tim* > *DmMANF-RNAi* flies (Supplementary Figure 6, Mann-Whitney Test, *p* = 0.153).

We also enquired if disturbed circadian rhythmicity were a consequence of impaired molecular mechanisms of the clock. Thus, we immunostained for PER and quantified its level in s-LNvs and l-LNvs at two time points in LD 12:12 and DD. Our results showed that DmMANF knockdown did not affect the cycling of PER in PDF-positive neurons in either condition (Supplementary Figure 7).

Our data argue that DmMANF is an important component for the correct development of the projections of the PDF-positive l-LNv and s-LNv neurons but it is not involved in the regulation of the molecular mechanisms of the circadian clock in these neurons.

### Morphology of the l-LNv Terminals in the Medulla Is Regulated by the *Drosophila melanogaster* Mesencephalic Astrocyte-Derived Neurotrophic Factor Protein in a Non-autonomous Manner

**FIGURE 5:**
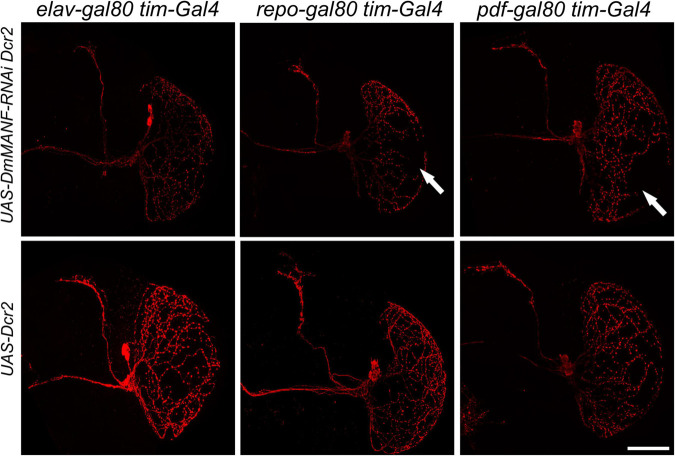
Morphology of PDF-expressing neurons after inhibition of DmMANF expression in *tim*-positive cells in combination with different *GAL80* lines. Morphology of PDF-positive neurons after expression of *UAS-DmMANF-RNAi* and Dicer2 (upper row) or Dicer2 alone as the control (bottom row) in *tim*-positive glia (left column), *tim*-positive neurons (middle column) and *tim*-positive cells excluding PDF-expressing pacemakers (right column). Solid arrows show the lack of l-LNv projections in the medulla. *N* = 9. Scale bar: 50 μm.

As mentioned above, in the adult brain DmMANF is expressed in neurons and glia. The *tim*-GAL4 driver targets central and peripheral clocks, including glial oscillators. Thus, we decided to identify the cells responsible for the abnormal neurite extension of the l-LNvs in the optic lobe. Using *tim-GAL4* and *UAS-DmMANF-RNAi, UAS-Dicer2* in combination with the repressors *repo-GAL80* and *elav-GAL80* we were able to inhibit the expression of this neurotrophic factor exclusively in *tim*-positive neurons or glia, respectively. We observed that DmMANF produced in neurons is crucial for the correct morphology of the l-LNv since neurites were affected after silencing this neurotrophic factor in neurons. In contrast, neurites remained intact after inhibition of DmMANF in *tim*-expressing glia (Figure 5). We also tested flies where *UAS-DmMANF-RNAi, UAS-Dicer2* are driven by *Pdf-GAL80*; *tim-GAL4* and found that the projections of the l-LNvs to the medulla were still affected (Figure 5). These data suggest that the morphology of the l-LNvs is controlled by DmMANF produced in other neurons or that the repression exerted by *Pdf-GAL80* is too weak to rescue the phenotypic effect.

**FIGURE 6:**
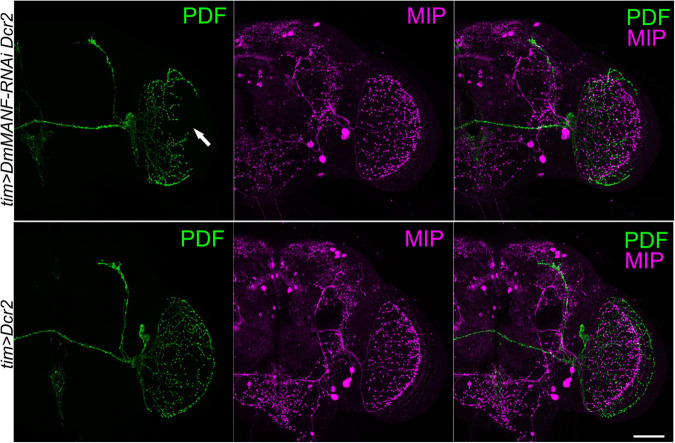
Double labeling of PDF-positive and MIP-expressing neurons in the head of flies with silenced expression of DmMANF in clock neurons. Brains of *tim > DmMANF-RNAi Dcr2* (upper panel) and *tim > Dcr2* (lower panel) were double-labeled with antiserum to PDF (green) and MIP (magenta). The morphology of PDF-positive neurons terminals in the medulla (arrow) is disturbed when DmMANF is silenced. Morphology of terminals of MIP-expressing neurons in the medulla remains intact. *N* = 7. Scale bar: 50 μm.

Finally, to test if DmMANF produced by *tim*-expressing cells alters neurite morphology of other cells that project to the medulla we used antibodies against myoinhibitory peptide (MIP) to visualize additional optic lobe neurons and their processes. A cluster of three large neurons is located in the lateral protocerebrum next to the medulla and accessory medulla and send projections to the medulla, the lamina, the dorsal part of the brain and neuropil in the lateral protocerebrum. The morphology of MIP-expressing neurons partly resembles that of PDF-expressing neurons; these cells do not express PDF and TIM, however (Kolodziejczyk and Nässel, 2011). *tim*-driven knockdown of DmMANF did not change the morphology of MIP-positive neurons, suggesting that DmMANF in *tim*-expressing neurons is important for the development of the l-LNv terminals in the medulla but not for neurites of other neurons in this part of the brain (Figure 6).

### The Lack of *Drosophila melanogaster* Mesencephalic Astrocyte-Derived Neurotrophic Factor in L2 Neurons in the Lamina Abolishes Their Daily Morphological Plasticity

**FIGURE 7:**
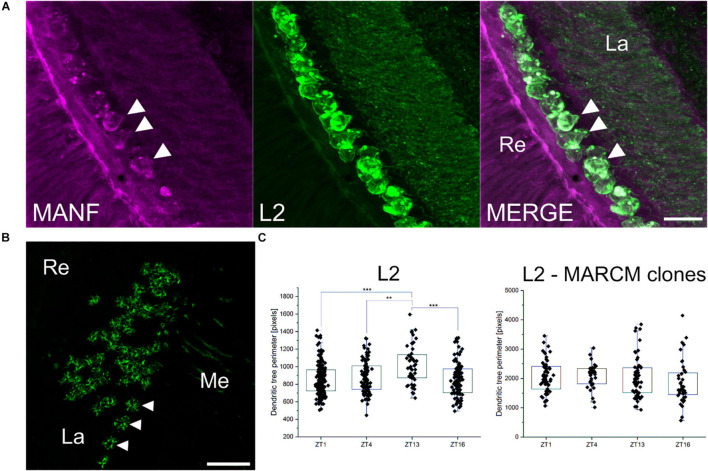
The role of DmMANF in L2 interneurons in the visual system. **(A)** Expression of DmMANF (magenta) in cell bodies of GFP-labelled L2 interneurons (green) in the lamina. Arrowheads indicate DmMANF-expressing L2 cells **(B)** Cross section of the lamina showing MARCM clones (L2 and their dendritic trees marked by GFP) mutant for *DmMANF*. Arrowheads indicate L2 neurons **(C)** The pattern of the daily rhythm of the L2 dendritic trees of *21D-GAL4 > UAS-mCD8::GFP* (*N* = 20–25) and MARCM clones mutant for DmMANF (*N* = 15–20). In the control group, the dendritic trees were largest at the beginning of the night. The rhythm was abolished in cells lacking DmMANF. Multiple comparison test, ***—*p* ≤ 0.001, **—*p* ≤ 0.01. Re: retina, La: lamina, Me: medulla. *N* represents the number of individuals for each analysis. Scale bars: 10 μm **(A)** and 20 μm **(B)**. Graphs represent median ± interquartile (the whiskers are determined by the 5th and 95th percentiles).

In the lamina, DmMANF is present not only in glial cells but also in cell bodies of L2 interneurons (Figure 7A). In our previous studies (Pyza and Meinertzhagen, 1999; Weber et al., 2009), we found that the L2 dendritic trees exhibit structural circadian plasticity, and their size and shape change during the day and night. The lack of DmMANF, in analysed MARCM strains (Figure 7B), abolished rhythmic plasticity of the L2 dendritic tree (Figure 7C). The L2 cell dendritic trees of control flies (*21D-GAL4 > UAS-mCD8::GFP*), which were measured in the distal lamina, showed daily rhythms in size and shape changes, with a peak at the beginning of the night (ZT13) [Kruskal-Wallis Test, *p* < 0.001 (Figure 7C)]. Interestingly, the dendritic trees without DmMANF were slightly larger, but also had a more complex structure (more branching) (Figure 7C and Supplementary Figure 9).

## Discussion

So far, only a few studies have examined the effects of neurotrophic factors on the circadian clock. Liang et al. (1998) have shown that in the rat suprachiasmatic nucleus BDNF mRNA and protein oscillate in a circadian fashion with peaks during the early subjective day and the subjective night, respectively. Additionally, this neurotrophic factor shows circadian changes in plasma concentration (Cain et al., 2017). It has been suggested that BDNF-mediated signalling may play an important role in the circadian regulation of SCN pacemaker sensitivity to light (Liang et al., 2000). Here, we have shown that another neurotrophic factor, DmMANF, which has been detected in *Drosophil*a (Palgi et al., 2009), is involved in the regulation of the circadian clock. In the brain of adult flies *DmMANF* is present in both neurons and glia (Stratoulias and Heino, 2015a), reaching particularly high levels in epithelial glial cells of the lamina (Walkowicz et al., 2017) and in two types of the medulla glia: astrocyte-like glia (AlGl) and ensheathing glia (EnGl) (Krzeptowski et al., 2018; Walkowicz et al., 2021). Both AlGl and EnGl express the core clock protein PERIOD (PER) and these glial subtypes have already been described as glial peripheral oscillators (Górska-Andrzejak et al., 2018; Krzeptowski et al., 2018).

In the present study, we showed that DmMANF is expressed in several clock cell clusters, including l-LNv and PDF-positive s-LNv, cryptochrome-positive LNd and DN1p neurons. Additionally, DmMANF was detected in *tim*-expressing cells in the second optic lobe, the medulla, including glial cells and neurons. Interestingly, DmMANF does not show circadian oscillations either in the whole head, or in the PDF-positive cells under both LD and DD conditions.

After silencing DmMANF in clock neurons using the *tim-GAL4* driver we found changes in the circadian rhythm of locomotor activity. Importantly, such changes do not seem caused by disruption of the molecular mechanism of the clock, since PER cycling in s-LNvs and l-LNvs was unaffected. Since even a single sLNv or lLNv neuron is sufficient to drive the rhythm of locomotor activity (Helfrich-Förster, 1998) we hypothesize that the lack of DmMANF in TIM-positive cells disrupts the circadian clock output pathways or it may be a consequence of decreased viability of those individuals. The impaired morning anticipation may result form altered morphology of PDF-positive terminals. This is supported by results of other authors showing that anticipatory activity is absent (Sheeba et al., 2010) 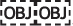. On the other hand, higher arrhythmicity in flies with silenced expression of *DmMANF* in *tim*-positive cells and the lengthening of period in DD may be caused by the disruption of the clock-controlled structural plasticity of the s-LNv terminals in the dorsal brain. The clock-controlled morphological changes of the s-LNvs involves remodelling of their axonal terminals in the dorsal brain. The s-LNv neurites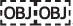. We observed that the s-LNv dorsal axonal arbors were less branched in DmMANF-deficient flies than in control flies. This is reminiscent of *per*^01^ and *tim*^01^ clock mutants, in which daily changes in the PDF circuit structure is also disrupted (Fernández et al., 2008).

Our results suggest that DmMANF, similarly to nerve growth factor and other members of the neurotrophin family, can regulate neurite outgrowth and support its function in axon guidance or growth and in branching of axon terminals (Cohen-Cory et al., 2010). MANF neurotrophic factor plays a conserved protective role for dopaminergic neurons, especially for the maintenance of their neurites (Petrova et al., 2003; Palgi et al., 2009). Importantly, microarray analysis showed that expression of genes involved in axon guidance, cell projection organization, neuron development or axonal defasciculation were affected in *DmMANF* mutants (Palgi et al., 2012). In flies with silenced *DmMANF* expression (*tim > DmMANF-RNAi Dcr2* individuals) we observed large changes in the morphology of PDF-positive terminals, both in the l-LNvs projections to the medulla and in the s-LNvs terminals in the dorsal protocerebrum. The knockdown of *DmMANF* in clock cells (in *tim-*positive cells) resulted in loss of normal arborisation of PDF-positive terminals in the medulla and reduction of s-LNvs terminal size. The number of cell bodies of l-LNv neurons was preserved, so the lack of projections in the medulla is not a consequence of cell degeneration. Interestingly, our results resemble the phenotype of flies with overexpression of *miR-210* in clock cells (Cusumano et al., 2018). The authors have shown that *miR-210* affects projections and shape of PDF-expressing neurons and modulates the circadian rhythm of locomotor activity of flies with no change in the PER cycling. Moreover, microarray analysis confirmed that *DmMANF* expression is downregulated in miR-210 over-expressing flies at ZT0 (Cusumano et al., 2018). It is worth to emphasize the fact that neither our results (Supplementary Figure 8) nor the study by Cusumano et al. (2018) showed morphological changes when the *Pdf-GAL4* driver line was used. It is possible that this driver line crossed to *DmMANF-RNAi* one gives only small effects in the targeted neurons which are not visible at the confocal microscope level, or morphological changes of the l-LNvs are only visible when DmMANF level is reduced in PDF neurons, other neurons and in neighbouring glial cells which all express TIM. Glial cells may affect clock neurons, for example by release of DmMANF which modulates neurite branching through DmMANF receptors. To our knowledge, the DmMANF receptor in *Drosophila* has not been described yet. However, in HeLA cells neuroplastin (NPTN) has been identified as a surface receptor for MANF as its inhibitory factor (Yagi et al., 2020). It is worth to notice that DmMANF in neurons, not in glial cells, is responsible for less developed morphology of the l-LNVs neuronal network (Figure 5). We have found (Krzeptowski et al., 2018) that in the distal medulla there are TIM-positive but REPO-negative cells, so they belong to neurons. Since these cells express DmMANF, they could regulate growth of the l-LNvs neurites. The lack of DmMANF in TIM-positive neurons may disturb chemotactic signals which are important for a proper guidance of the l-LNv projections in the medulla during development. In our experiments morphological changes were observed only when *DmMANF* silencing was enhanced by the overexpression of Dicer2, a protein that cleaves pre-miRNAs to mature miRNAs. However, we did not observe phenotypic effect in a control line in which Dicer2 protein was expressed alone, which indicates that the changes were related to the DmMANF deficiency.

Interestingly, our other studies showed that overexpression of DmMANF in astrocyte-like glia results in abnormal arborisation of PDF-expressing neurons (Walkowicz et al., 2021). The finding rises the question of whether the morphological changes of PDF-positive neurons are related to disturbances in the expression of DmMANF specifically in *tim*-positive glia. To test this hypothesis we used the GAL80 repressor and found that the effect observed after *DmMANF* silencing was restricted to neurons. We also confirmed that the silencing of DmMANF in clock cells affects the morphology of PDF-positive neurons specifically. We did not detect abnormal arborisation in the medulla of MIP-expressing neuron. We examined those cells because their morphology partly resembles that of PDF-positive neurons (Kolodziejczyk and Nässel, 2011).

Finally, we found that lack of DmMANF leads to abnormal morphology also in L2 monopolar cells. These cells are a well-known model for studying circadian plasticity (Pyza, 2013). The L2 dendrites are longer at the beginning of the day and night when tetrad synapses between photoreceptors and four post-synaptic cells, including L2, are most numerous and L2 axons are swollen. These changes are controlled by the circadian clock (Pyza and Meinertzhagen, 1999; Weber et al., 2009; Woźnicka et al., 2015; Kijak and Pyza, 2017). The lack of DmMANF in L2 neurons abolished the daily changes in morphology of their dendritic tree. A constant turnover of the plasma membrane of neurons, which is needed for maintaining the morphology and the remodelling of the dendritic tree, is mediated primarily by endocytic and secretory pathways (Chung et al., 2020). Microarray studies have shown that lack of DmMANF results in downregulation of several genes in the exocytosis pathway (Palgi et al., 2012). Thus, we suspect that the changes in L2 morphology are a consequence of impaired membrane turnover. Moreover, the L2 dendritic trees without DmMANF were slightly larger, but mainly had a more complex structure. The lack of DmMANF in L2 cells may also result in impaired development of dendrites. A similar effect was observed in *per*^0^ mutants, but in contrast L2 dendrites were shorter than in wild-type flies (Weber et al., 2009).

In conclusion, our results suggest that DmMANF regulates the development of PDF-positive clock neurites but it is also important for maintaining circadian plasticity of non-clock neurons in the fly’s visual system.

## Data Availability Statement

The raw data supporting the conclusions of this article will be made available by the authors, without undue reservation.

## Author Contributions

WK and LW carried out experiments, analysed data, prepared figures, and wrote the first draft of the manuscript. EK, EM, KW, JS, and TA helped in experiments. ZB and ZR helped in confocal imaging. ER, VS, and TH provided transgenic strains. ER and TH corrected the first draft of the manuscript. EP analysed results, proposed the concept of the manuscript, wrote the final version of the manuscript, and provided funding. All authors approved the manuscript.

## Conflict of Interest

The authors declare that the research was conducted in the absence of any commercial or financial relationships that could be construed as a potential conflict of interest.

## Publisher’s Note

All claims expressed in this article are solely those of the authors and do not necessarily represent those of their affiliated organizations, or those of the publisher, the editors and the reviewers. Any product that may be evaluated in this article, or claim that may be made by its manufacturer, is not guaranteed or endorsed by the publisher.

## Supplementary Material

Supplementary Figure 1 The level of DmMANF in the retina. The level of DmMANF in whole head homogenates in Canton S control flies and in flies with reduced eyes (EGUF/+; FRT80B GMR-hid3L/TM6B). The anti-DmMANF Ab labels a band at 19 kDa. α-Tubulin was used as a loading control.

Supplementary Figure 2 Rhythmic expression of DmMANF in the head. **(A)** Western blot (left panel) showing the level of DmMANF in head homogenates collected at ZT1, ZT4, ZT13, and ZT16. Densitometric analysis of DmMANF (right panel) was normalized to α-tubulin, used as a loading control (*N* = 8). L - protein ladder. **(B)** Western blot (left panel) showing the level of DmMANF in head homogenates collected at CT1, CT4, CT13, and CT16 of DD. Densitometric analysis of DmMANF (right panel) was normalized to α-tubulin, used as a loading control (*N* = 3). L - protein ladder. Graphs represent median ± interquartile (the whiskers are determined by the 5th and 95th percentiles). **(C)** Data quantification by measuring pixels intensity for DmMANF in s-LNvs and l-LNvs at ZT1, ZT4, ZT13, and ZT16. (Kruskal Wallis, *p* = 0.357, *N* = 115 and *p* = 0.377, *N* = 120, for s-LNVs and l-LNVs, respectively) Graphs represent mean ± SD.

Supplementary Figure 3 Expression of DmMANF in the head **(A)** Western blot showing the level of DmMANF in head homogenates of *tim/+* (lane 1), *+/DmMANF-RNAi* (lane 2), *tim > DmMANF-RNAi* (lane 3), *tim > Dcr2* (lane 4), *+/DmMANF-RNAi Dcr2* (lane 5), *tim > DmMANF-RNAi Dcr2* (lane 6). L - protein ladder. **(B)** Densitometric analysis of DmMANF was normalized to α-tubulin used as a loading control.

Supplementary Figure 4 Actograms of *tim* > Dm*MANF-RNAi Dcr2, tim > Dcr2, and DmMANF-RNAi Dcr2/+* flies.

Supplementary Figure 5 Numbers of l-LNvs cell bodies of flies with a decreased level of DmMANF in clock neurons. The analysis of the number of PDF positive cell bodies of *tim > DmMANF-RNAi Dcr2* insects showed no statistically significant differences compared to the *tim > Dcr2* control group (Mann-Whitney Test, *p* = 0.15). *N* = 24–36. Graphs represent mean ± SD.

Supplementary Figure 6 Sholl analysis of s-LNv projections of *tim* > *DmMANF-RNAi Dcr2* flies. Sholl analysis shows no differences (Mann-Whitney Test, *p* = 0.153) of total axonal crosses of *tim* > *DmMANF-RNAi* flies compared to *tim/+* flies. Graphs represent mean ± SD.

Supplementary Figure 7 PER expression in s-LNv and l-LNvs of flies with a decreased level of DmMANF in clock neurons. **(A)** Double labeling of PER and PDF of whole mount brains dissected at ZT1 and ZT14 time points showed that in both s-LNvs (arrows) and l-LNvs (arrowheads) of *tim* > *DmMANF-RNAi Dcr2* flies and control group PER protein is cycling. **(B)** Data quantification by measuring pixels intensity for PER channel showed statistical differences of PER fluorescence between timepoints in experimental and control flies bot in l-LNvs and s-LNvs (*t*-test with Welch’s correction, *p* < 0.001). **(C)** In DD PER level cycle in s-LNvs (arrows) but not in l-LNvs (arrowheads). **(D)** Fluorescence quantification of PER protein level in different timepoints in DD showed statistically significant changes for s-LNvs in experimental and control groups (*t*-test witch Welch’s correction, *p* < 0.0001) and no changes for l-LNvs (*t*-test witch Welch’s correction, *p* = 0.70 and *p* = 0.05). *N* represents the number of individuals for each analysis. Means ± SD, ^∗∗∗^ - *p* < 0.01. Me – medulla. Scale bar: 20 μm.

Supplementary Figure 8 Characterization of flies with silenced expression of *DmMNAF* under *pdf-Gal4* control. (Upper panel) PDF immunostaining of the projections of the s-LNvs in the dorsal brain and of the l-LNvs in the medulla. Flies with reduced DmMANF expression (pdf > *DmMANF-RNAi* and pdf > *DmMANF-RNAi Dcr2*) are compared to the control (*pdf*/+).

Supplementary Figure 9 Cross sections of the L2 axon with dendrites in wild-type flies and MARCM clones.
