# Supplementary figures and images for: Mesencephalic Astrocyte-Derived Neurotrophic Factor Regulates Morphology of Pigment-Dispersing Factor-Positive Clock Neurons and Circadian Neuronal Plasticity in Drosophila melanogaster
Source: Front Physiol. 2021 Sep 27;12:705183. doi: 10.3389/fphys.2021.705183 (PMC8502870; doi:10.3389/fphys.2021.705183)

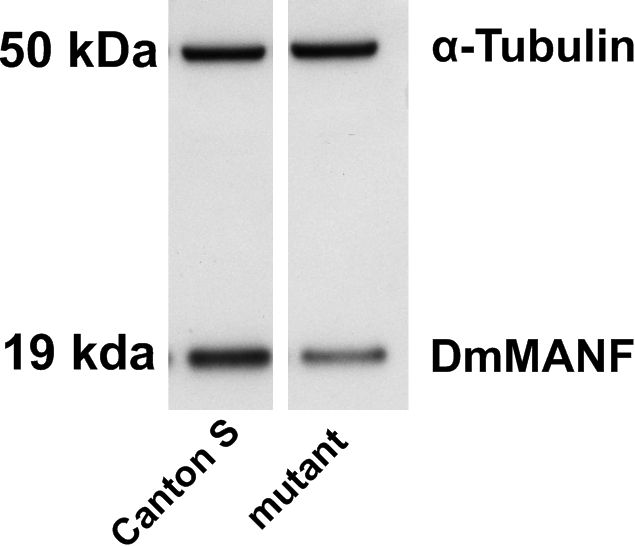

Supplement: Supplementary Figure 1 — The level of DmMANF in the retina. The level of DmMANF in whole head homogenates in Canton S control flies and in flies with reduced eyes (EGUF/+; FRT80B GMR-hid3L/TM6B). The anti-DmMANF Ab labels a band at 19 kDa. α-Tubulin was used as a loading control. [file Image_1.JPEG]

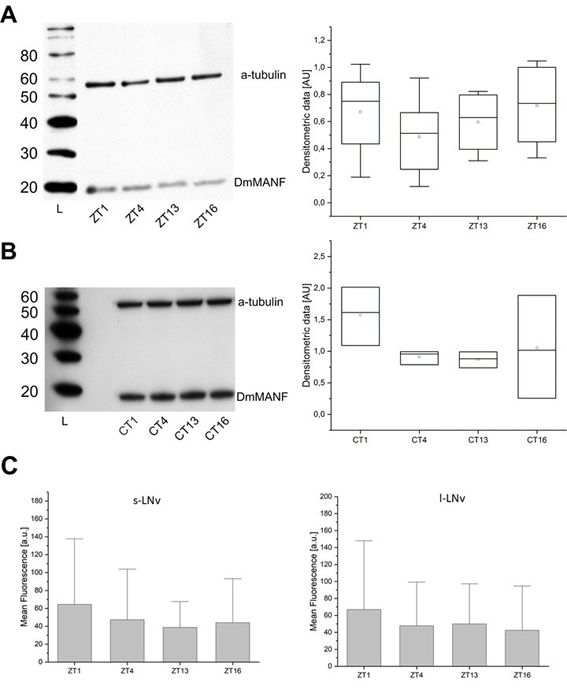

Supplement: Supplementary Figure 2 — Rhythmic expression of DmMANF in the head. (A) Western blot (left panel) showing the level of DmMANF in head homogenates collected at ZT1, ZT4, ZT13, and ZT16. Densitometric analysis of DmMANF (right panel) was normalized to α-tubulin, used as a loading control (N = 8). L - protein ladder. (B) Western blot (left panel) showing the level of DmMANF in head homogenates collected at CT1, CT4, CT13, and CT16 of DD. Densitometric analysis of DmMANF (right panel) was normalized to α-tubulin, used as a loading control (N = 3). L - protein ladder. Graphs represent median ± interquartile (the whiskers are determined by the 5th and 95th percentiles). (C) Data quantification by measuring pixels intensity for DmMANF in s-LNvs and l-LNvs at ZT1, ZT4, ZT13, and ZT16. (Kruskal Wallis, p = 0.357, N = 115 and p = 0.377, N = 120, for s-LNVs and l-LNVs, respectively) Graphs represent mean ± SD. [file Image_2.JPEG]

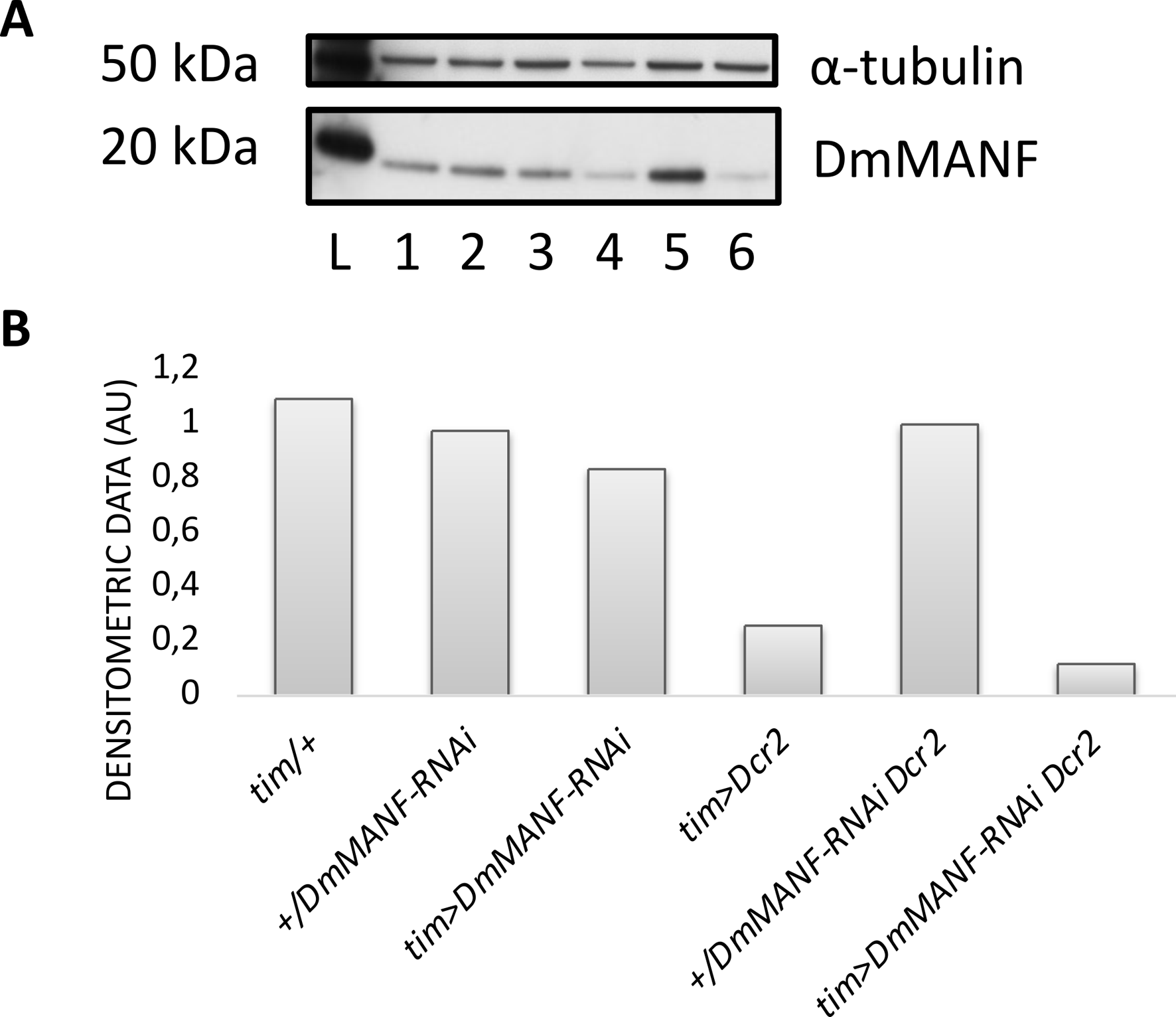

Supplement: Supplementary Figure 3 — Expression of DmMANF in the head (A) Western blot showing the level of DmMANF in head homogenates of tim/+ (lane 1), +/DmMANF-RNAi (lane 2), tim > DmMANF-RNAi (lane 3), tim > Dcr2 (lane 4), +/DmMANF-RNAi Dcr2 (lane 5), tim > DmMANF-RNAi Dcr2 (lane 6). L - protein ladder. (B) Densitometric analysis of DmMANF was normalized to α-tubulin used as a loading control. [file Image_3.TIF]

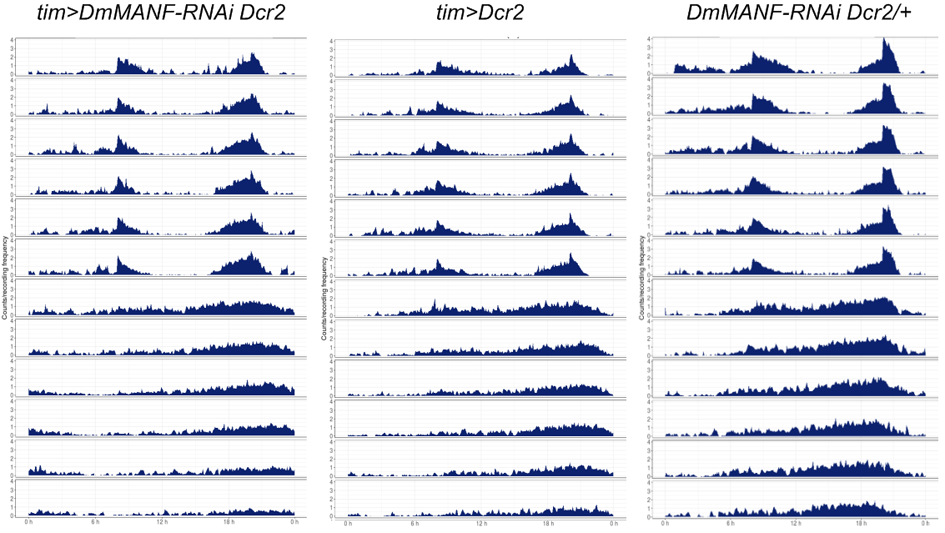

Supplement: Supplementary Figure 4 — Actograms of tim > DmMANF-RNAi Dcr2, tim > Dcr2, and DmMANF-RNAi Dcr2/+ flies. [file Image_4.TIF]

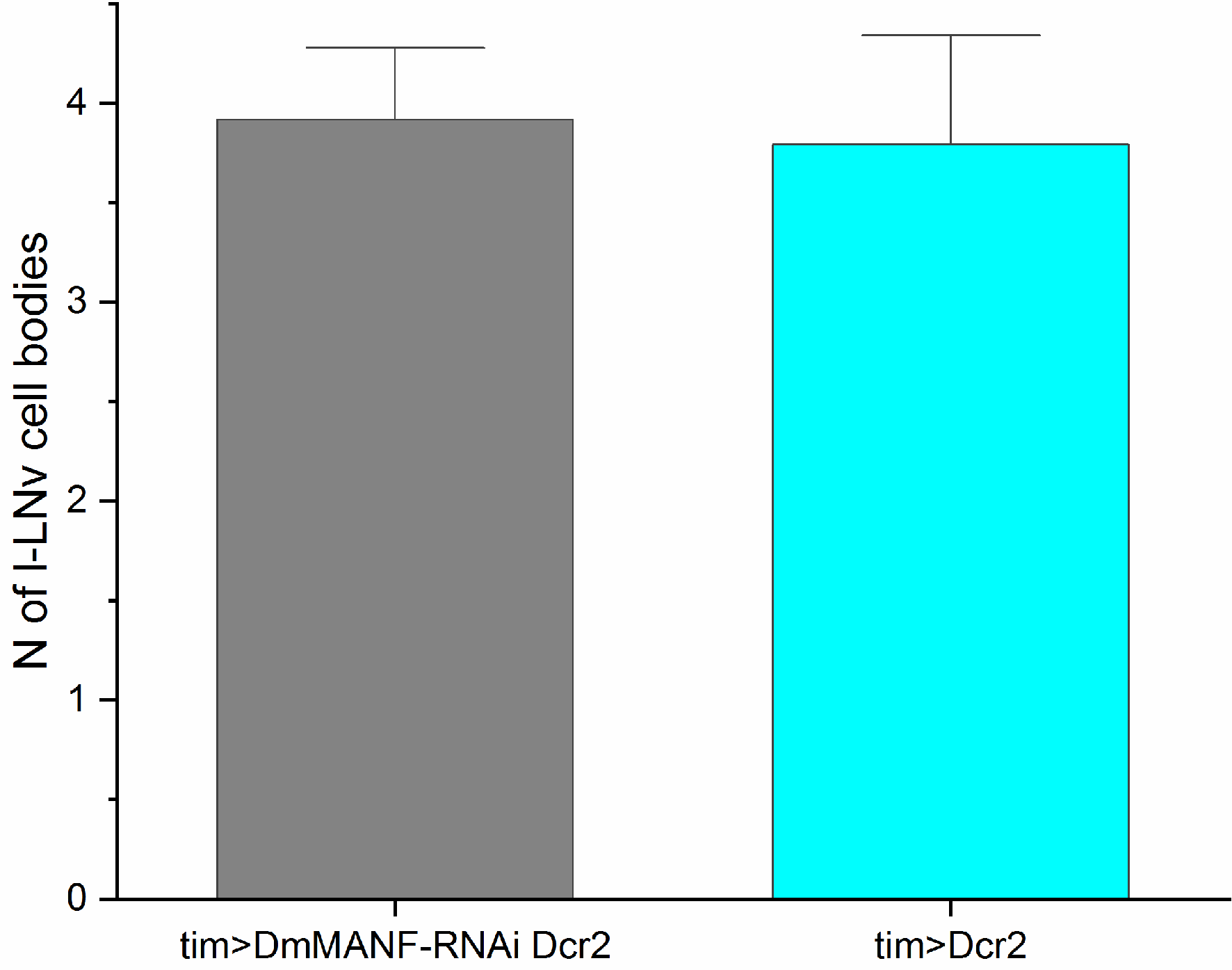

Supplement: Supplementary Figure 5 — Numbers of l-LNvs cell bodies of flies with a decreased level of DmMANF in clock neurons. The analysis of the number of PDF positive cell bodies of tim > DmMANF-RNAi Dcr2 insects showed no statistically significant differences compared to the tim > Dcr2 control group (Mann-Whitney Test, p = 0.15). N = 24–36. Graphs represent mean ± SD. [file Image_5.TIF]

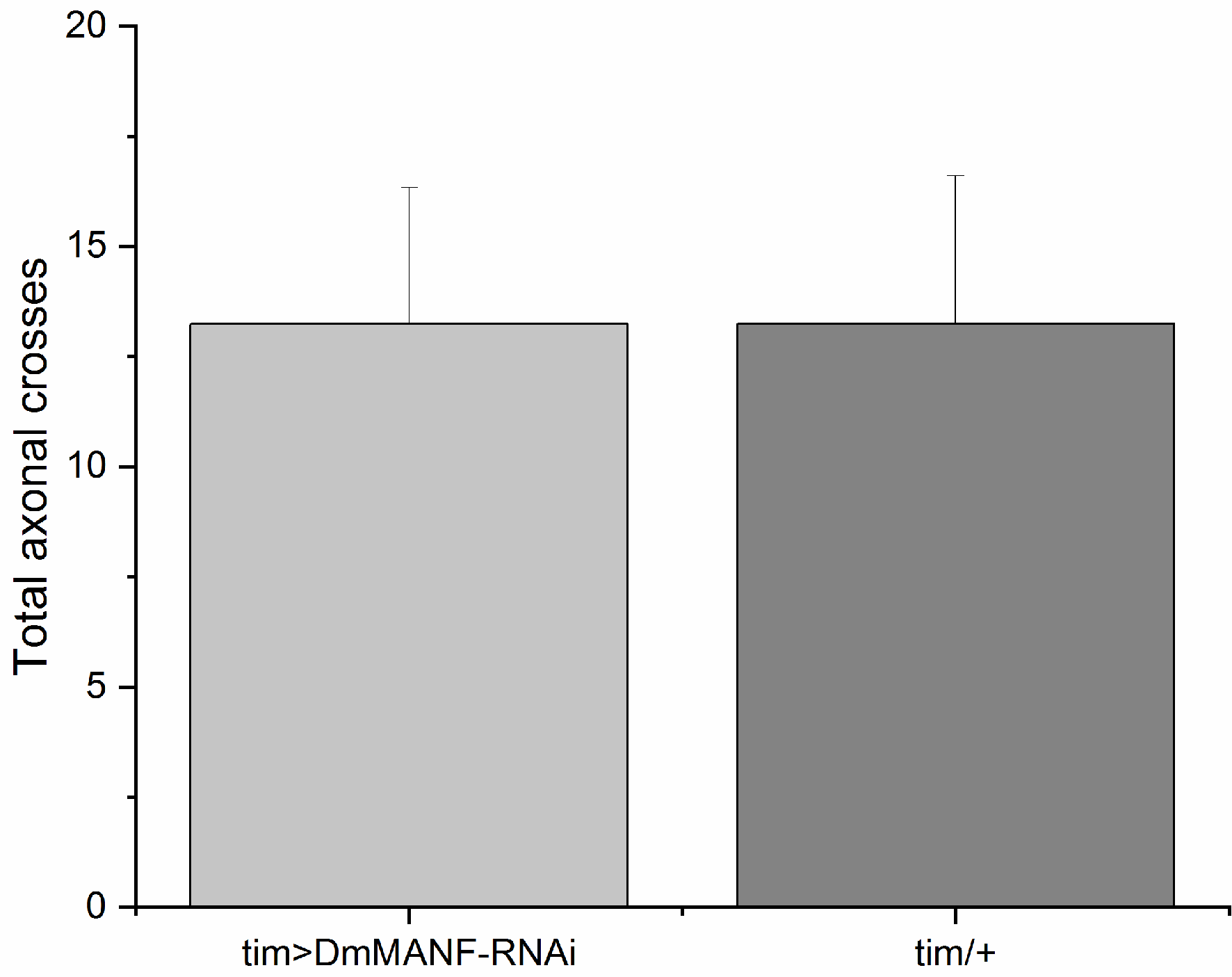

Supplement: Supplementary Figure 6 — Sholl analysis of s-LNv projections of tim > DmMANF-RNAi Dcr2 flies. Sholl analysis shows no differences (Mann-Whitney Test, p = 0.153) of total axonal crosses of tim > DmMANF-RNAi flies compared to tim/+ flies. Graphs represent mean ± SD. [file Image_6.TIF]

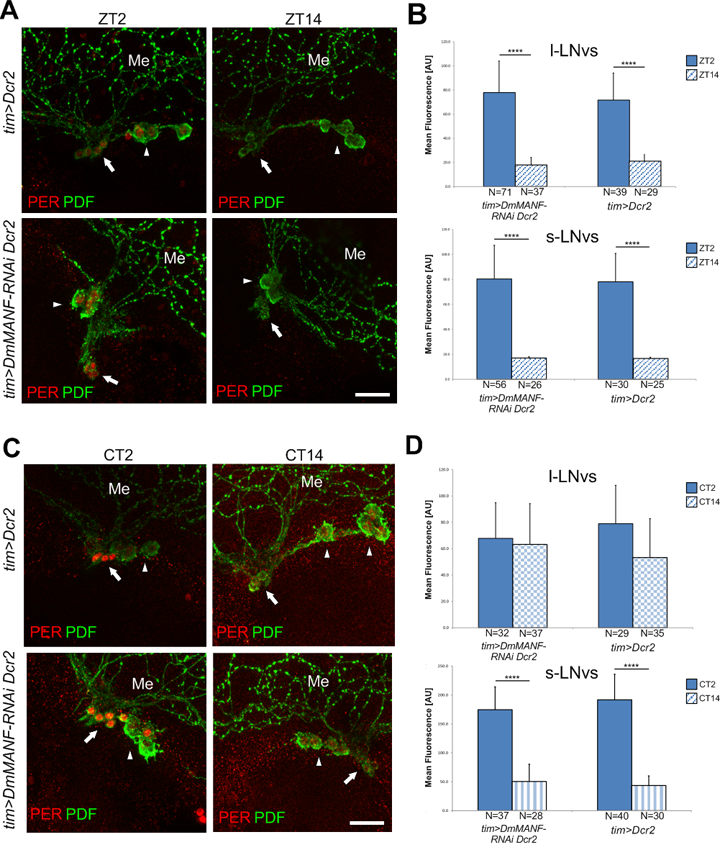

Supplement: Supplementary Figure 7 — PER expression in s-LNv and l-LNvs of flies with a decreased level of DmMANF in clock neurons. (A) Double labeling of PER and PDF of whole mount brains dissected at ZT1 and ZT14 time points showed that in both s-LNvs (arrows) and l-LNvs (arrowheads) of tim > DmMANF-RNAi Dcr2 flies and control group PER protein is cycling. (B) Data quantification by measuring pixels intensity for PER channel showed statistical differences of PER fluorescence between timepoints in experimental and control flies bot in l-LNvs and s-LNvs (t-test with Welch’s correction, p < 0.001). (C) In DD PER level cycle in s-LNvs (arrows) but not in l-LNvs (arrowheads). (D) Fluorescence quantification of PER protein level in different timepoints in DD showed statistically significant changes for s-LNvs in experimental and control groups (t-test witch Welch’s correction, p < 0.0001) and no changes for l-LNvs (t-test witch Welch’s correction, p = 0.70 and p = 0.05). N represents the number of individuals for each analysis. Means ± SD, ∗∗∗ - p < 0.01. Me – medulla. Scale bar: 20 μm. [file Image_7.TIF]

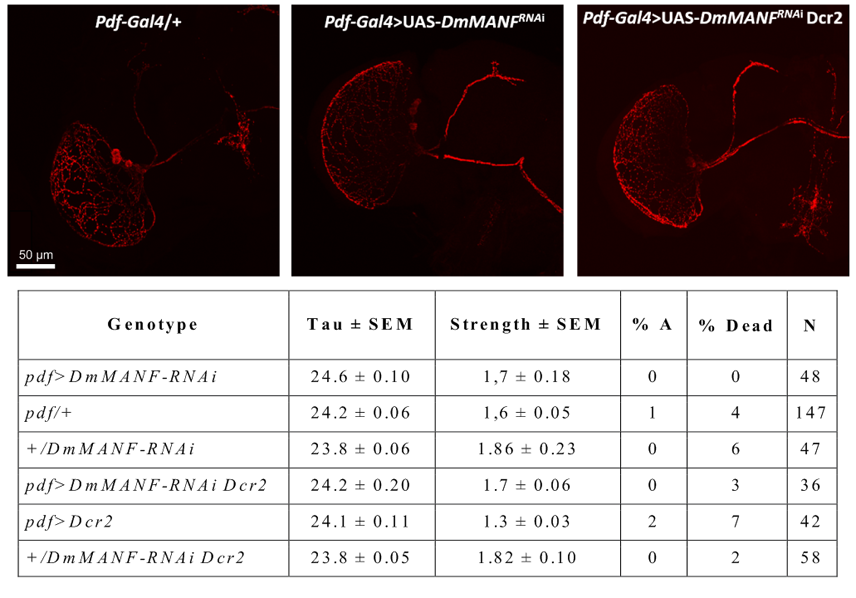

Supplement: Supplementary Figure 8 — Characterization of flies with silenced expression of DmMNAF under pdf-Gal4 control. (Upper panel) PDF immunostaining of the projections of the s-LNvs in the dorsal brain and of the l-LNvs in the medulla. Flies with reduced DmMANF expression (pdf > DmMANF-RNAi and pdf > DmMANF-RNAi Dcr2) are compared to the control (pdf/+). [file Image_8.TIF]

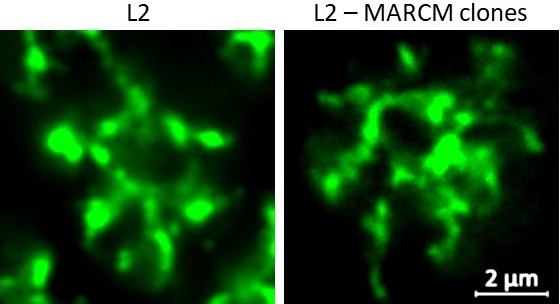

Supplement: Supplementary Figure 9 — Cross sections of the L2 axon with dendrites in wild-type flies and MARCM clones. [file Image_9.JPEG]
